# Potential Use of Microbial Community Genomes in Various Dimensions of Agriculture Productivity and Its Management: A Review

**DOI:** 10.3389/fmicb.2022.708335

**Published:** 2022-05-17

**Authors:** Mir Asif Iquebal, Jaisri Jagannadham, Sarika Jaiswal, Ratna Prabha, Anil Rai, Dinesh Kumar

**Affiliations:** ^1^Centre for Agricultural Bioinformatics, ICAR-Indian Agricultural Statistics Research Institute, New Delhi, India; ^2^School of Interdisciplinary and Applied Sciences, Central University of Haryana, Mahendergarh, Haryana, India

**Keywords:** agriculture, metagenomics-wide association studies, metagenome, software tools, web resources

## Abstract

Agricultural productivity is highly influenced by its associated microbial community. With advancements in omics technology, metagenomics is known to play a vital role in microbial world studies by unlocking the uncultured microbial populations present in the environment. Metagenomics is a diagnostic tool to target unique signature loci of plant and animal pathogens as well as beneficial microorganisms from samples. Here, we reviewed various aspects of metagenomics from experimental methods to techniques used for sequencing, as well as diversified computational resources, including databases and software tools. Exhaustive focus and study are conducted on the application of metagenomics in agriculture, deciphering various areas, including pathogen and plant disease identification, disease resistance breeding, plant pest control, weed management, abiotic stress management, post-harvest management, discoveries in agriculture, source of novel molecules/compounds, biosurfactants and natural product, identification of biosynthetic molecules, use in genetically modified crops, and antibiotic-resistant genes. Metagenomics-wide association studies study in agriculture on crop productivity rates, intercropping analysis, and agronomic field is analyzed. This article is the first of its comprehensive study and prospects from an agriculture perspective, focusing on a wider range of applications of metagenomics and its association studies.

## Introduction

Metagenomics (community and environmental genomics), once described as a major demand in microbial analysis, is uncovered through methods that drive microbial community population studies on heterogeneity and complexity over time. The goal for any metagenomic study is to completely characterize a microbial community, *i.e.*, “who’s present?”, “what are their actions?”. This helps to decipher the structure of the microbial community, the functional activity of each microbial member, and the intra-species heterogeneity information (Scholz et al., 2012).

The term “metagenomics” was coined in 1998 to capture the analysis knowledge of a set of similar but not identical organisms, as in meta-analysis, which is defined as the analysis of analyses (Handelsman et al., 1998). Stepwise advancement in metagenomics study includes proposal involving the cloning of DNA from environmental-related samples, phage vector study, and construction of metagenomic library from DNA. The metagenomics field was initially defined by constructing the libraries from prokaryotic organisms in seawater with 16S rRNA (Schmidt et al., 1991; Stein et al., 1996). Later, similar studies were reported using 16S rRNA from soil samples (Henne et al., 1999). Soil is considered the most challenging environment due to the size and diversity of the microbiome (Daniel, 2005). A study estimated one gram of microbial population in forest soil containing 4 × 10^7^ prokaryotic cells (Richter and Markewitz, 1995) while cultivated soils and grasslands contained an estimated 2 × 10^9^ prokaryotic cells (Paul and Clark, 1989). Later on, ‘metagenomic’ was termed as ‘the functional and sequence-based analysis of the collective microbial genomes of an environmental sample’ (Allen and Banfield, 2005; Sarangi et al., 2019).

The revolution of metagenomics analysis started with computational technology and advancement in sequencing. The decreasing cost of next-generation sequencing and increase in analytical applications have led to a major transformation in the field of the microbial world. Microbial community characterization is progressive using various sequencing methods, namely, Sanger, pyrosequencing, ABI-solid, 454, Illumina, assembled, and so on (Table 1). Metagenomics involves DNA extraction and cloning from a collection of microorganisms. It is emanated from inevitable studies that microorganisms represent a massive population probably in all environments on earth especially 16S rRNA analyses approach facilitates the detection of ample new microbial life lineages (Dhanjal et al., 2020a). Though 16s rRNA revolutionized the detection of the microbial community, metagenomics has led to a detailed study on environmental microorganisms in the context of ecology and physiology.

**TABLE 1:** Different next-generation sequencing platforms used in metagenomics sequencing.

Company	Platforms	Read length (bp)	Run time (hours)	Output – throughput per run (GB)	Web link
ABI-Sanger	3500 genetic analyzer	500–900	0.5–2.5	Differ on polymer type	https://www.thermofisher.com/order/catalog/product/4359571#/4359571
Illumina	MiSeq	36–250	39	8.5	https://www.illumina.com/
	HiSeq 2500	36–150	264	600	
Roche	Genome Sequencer (GS) FLX Titanium	1000	23	1	Roche: https://www.roche.com
	GS Junior System	500	10	0.035	
Life Technologies	Proton	200	4	10	https://www.thermofisher.com/in/en/home/brands/life-technologies.html
	Ion Torrent with Personal Genome Machine (PGM) 318 Chip	400	7	2	
Oxford Nanopore	MinION	48,000	Differ	Differ	−
Pacific Biosystems	PacBio RS	2,000–15,000	2	0.1	https://www.pacb.com/

**FIGURE 1:**
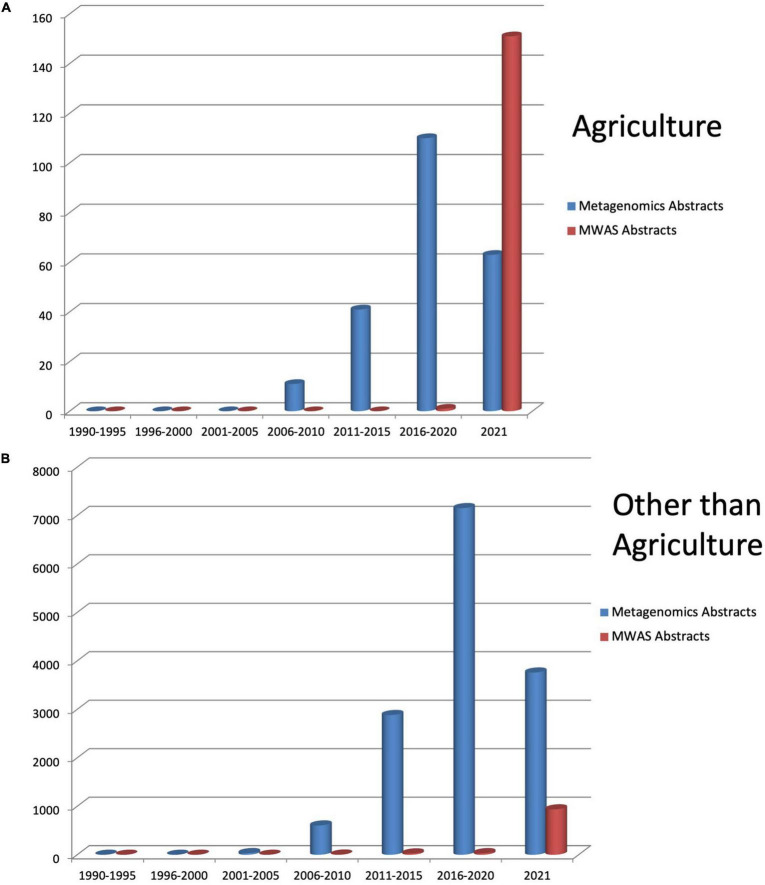
Literature reported in PubMed for metagenomics and metagenomics wide association studies from 1990 till 2021 in **(A)** agriculture and **(B)** other than agriculture.

The need for metagenomics has gained significant concern among microbiologists due to earlier microbial techniques. It involves the growth of microbes in pure culture in the laboratory due to which many organisms might have missed or gone unnoticed during culturing. This cultivation bottleneck is overcome by metagenomics analysis by providing a relatively unbiased view of species richness, diversity, and their potential activity in the community population (Links et al., 2012). In general, a powerful combination of genome sequencing and bioinformatics analysis of data has transformed our understanding and knowledge about how organisms evolve, function, and interact with each other, their hosts, and with the environment, providing new channels of inquiries and advances for translational impact (Moreno-Indias and Tinahones, 2020). Garrido-Cardenas and Manzano-Agugliaro’s 2017 article on worldwide research in metagenomics shows an exponential rise every year in the study and application of metagenomics approaches from the beginning of the metagenomics era (Figure 1).

Metagenomics mainly deals with the analysis of environmental samples from diversified sources like agriculture and seawater. The environment is a major reservoir of microbial species diversity and the complexity of this diversity depends on various factors like pH, temperature, water content, biotic activity, soil structure, and climatic variations. Although microbial communities are key players in the functioning of all ecosystems, their identification and understanding role of uncultivable microorganisms in natural ecosystems are uncertain (Tyson et al., 2004). Also, metagenomics helps in unlocking the uncultured microbial population present in an environment, especially, novel molecules related to therapeutic, biotechnological, and sustainable agricultural applications. With this versatility, in this review, we present its wider applications in agriculture, including livestock health and disease concerns, crop production and effect of environmental factors, such as abiotic stress, novel products identified through this approach, and its industrial applications.

### Spatial Dimensions of Metagenomic Studies in the Biosphere

Metagenomics study is not limited to air (Núñez et al., 2016), water samples like aquatic (Doxey et al., 2015), freshwater (Edge et al., 2020), soil (Delmont et al., 2011), gut microbiota (Zhou et al., 2020), coral (Meenatchi et al., 2020), terragenome (Vogel et al., 2009), marine (Woyke et al., 2009), and oil-contaminated sediments from the Deepwater Horizon spill (Kimes et al., 2013). Based on human gut microbiota studies through metagenomics, a database atlas on gutMEGA was developed by Zhou et al. (2020). Various studies related to mass bleaching to understand the global impact on coral microorganisms (Meenatchi et al., 2020), iron intake trends by microbes prevailing on the surface ocean (Toulza et al., 2012; Meenatchi et al., 2020), ecobiomics projects to estimate and check soil health and quality of water (Edge et al., 2020), virus diversity and host interactions (Obiol et al., 2020; Schulz et al., 2020) have been undertaken. Recently, a study identified 1200 metagenome-assembled genomes from African cattle rumen and provided wider insights on the rumen functionality at extremely harsh conditions like food scarcity (Wilkinson et al., 2020). Another work estimated about 400 novel species and their ecological preferences in freshwater ecosystems (Rodriguez-R et al., 2020).

**FIGURE 2:**
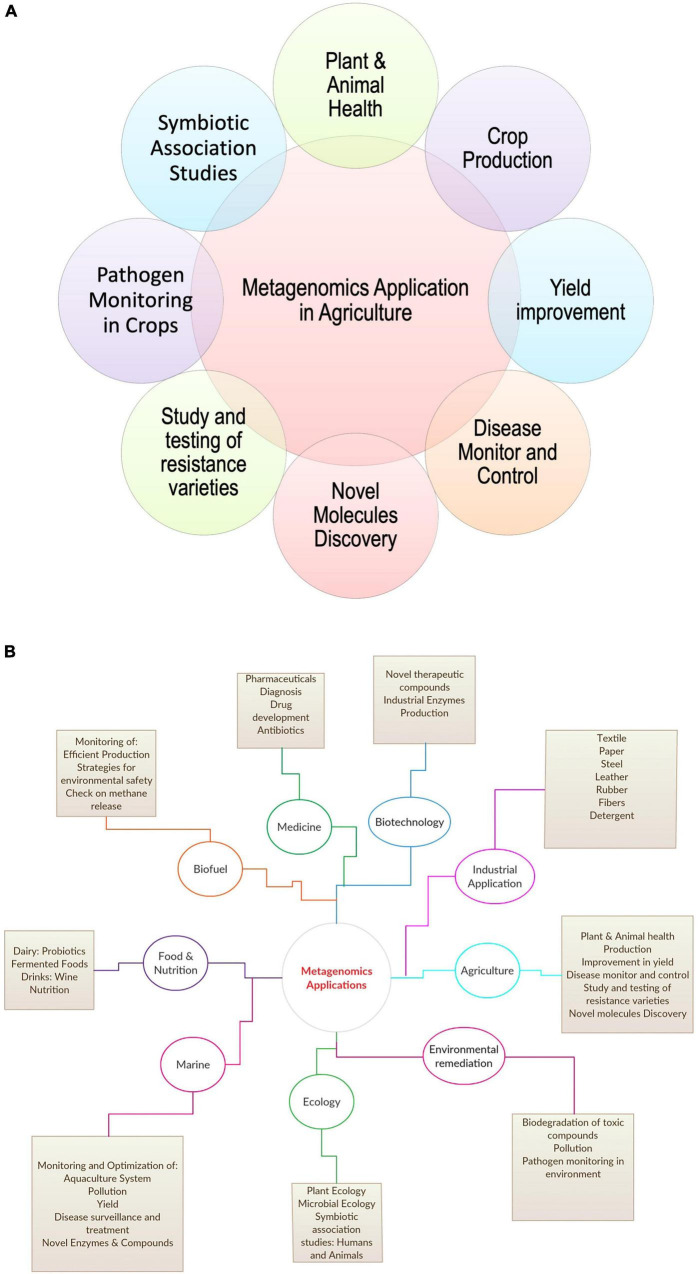
Application of metagenomics in **(A)** agriculture and **(B)** other areas.

A recent study by Kwok et al. (2020) on zoonotic origin infectious diseases, stated the role of metagenomic Next-Generation Sequencing (mNGS) in identifying and characterizing novel etiologies and viruses from diversified samples in livestock, such as pig, cattle, poultry, and small ruminants (Kwok et al., 2020). Further, recent studies on human microbiota in disease association stated the impact and importance of microbial ecology of livestock in achieving better food quality and a healthy environment for the host (Frank, 2011). Features, such as plant fitness, soil biogeochemical properties, quality traits, and crop yield, play a key role in agriculture depend majorly on the microbiome of soil, plant, and livestock (Esposito et al., 2016). Miller et al. (2013) elaborated on the role of metagenomics in the public health sector for new pathogen detection (Miller et al., 2013). The study also commented on constantly evolving and zoonotic in origin microorganisms like H7N9 influenza and Middle East respiratory syndrome coronavirus (MERS-CoV). All these studies show the broad application of metagenomics in agriculture and other sectors (Taş et al., 2021; Figure 2).

### Various Approaches and Tools for Metagenomics Data Analyses

Next-generation sequencing (NGS) is a powerful diagnostic tool in agriculture metagenomics due to its scope to target various unique signature loci of plant and animal pathogens as well as beneficial microorganisms from samples. The various approaches used in metagenomic studies include:

#### Amplicon-Based Methods

These are read-based reconstructions of the functional and taxonomic components of the metagenome. It is a targeted strategy, which involves a pre-sequencing PCR amplification to selectively target a taxonomic marker rRNA gene. Amplicon sequences are PCR products that specify a region within a gene and are specific to a particular organism. Strategies used through this method in bacterial characterization are 16S rRNA, RNA polymerase and heat shock protein 90, protozoan, and fungal identification with 18s rRNA gene (Iliev et al., 2012). In the case of viral population studies, viral RNA polymerase is used. In deep sequencing with next-generation technology, multiple different amplicons can be sequenced in a sample These sequences are assembled to a reference database to identify the conserved gene of a specific organism. This method identifies the organism along with its relative abundance. Overall, the sensitivity of this method increases in strain identification that is targeted to a particular species through amplicons. Though potential bias does exist in this method, such as the abundance ratio, due to artificially inflated counts of certain taxa during PCR amplification or with the use of universal primers which may mislead and may ignore the identification of certain organisms (Gonzalez et al., 2012).

#### Shotgun Metagenomics

It is a broader strategy that involves entire genome sequencing present in an environmental sample which facilitates a better understanding of the functions along with taxonomic profiling of the microbes (Enagbonma et al., 2019). The sequenced reads are compared to the reference database which is different and large in comparison with amplicon-based methods. This method is computationally intense, less biased, and reflects the better microbial community structure in a sample (Shakya et al., 2013). Moreover, this method has the power to differentiate the closely related species or strains (Miller et al., 2013). This method can also be used in the identification of stable microbial community structures in agricultural soil as well as novel ammonia oxidizers in fertilization (Orellana et al., 2018).

**FIGURE 3:**
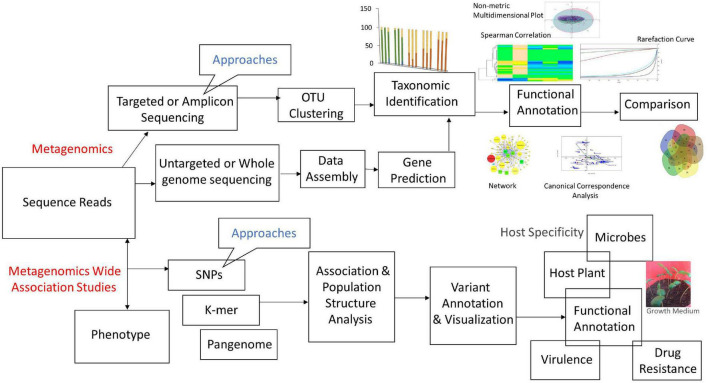
Flow-chart of metagenomics approaches and their association studies.

The general workflow of metagenomics includes: (i) processing of biological samples after sample collection and DNA/RNA extraction, (ii) sequencing, and (iii) bioinformatics analyses using various tools. Each step involving various approaches and tools is discussed precisely (Figure 3).

#### Processing of Biological Samples

Based on the purpose of the study, metagenomic analysis involves the extraction of the entire nucleic acid from a sample. Depending on the strategy, either DNA or RNA is targeted based on the microbial population to be studied, like, bacteria or viruses. In the case of virus studies in metagenomics, virions are extracted by removing cellular material followed by treatment of nucleases to remove non-viral nucleic acids and to extract viral nucleic acids intact within the nucleocapsid (Thurber et al., 2009). Technical issues related to very fewer amounts of nucleic acid in a sample can also be handled with library preparation kits for metagenomics, such as Nextera XT (Illumina, San Diego, CA, United States), random PCR, multiple displacement amplification, and higher sequencing depth (Loman et al., 2013).

Another important aspect in metagenomics is the collection of environmental sample associated data, i.e., metadata, that include temperature, pH, and salinity, as well as the collection of geographical data, such as global positioning system coordinates, depth, height, date of sample collection, extraction method, and clone library information (Field et al., 2008). Similarly, metadata with clinical samples varies like height, weight, sex, age, symptoms, heredity diseases, and so on. Databases for metagenomics also include the diverse degrees of metadata and also facilitate comparative communities study along environmental, longitudinal, or spatial gradients (Seshadri et al., 2007).

#### Sequencing

Sequencing is a crucial step that depends on various factors, such as samples being sequenced, sample size and cost. Various sequencing platforms available are discussed in Table 1. The most commonly used sequencing platforms in metagenomics analysis are Illumina and 454 pyrosequencing.

#### Bioinformatics Analysis

The computational analysis depends on sequencing methods, which use two main approaches in genome reconstruction: (i) Assembling of reads, followed by taxonomic and functional classification: Shotgun sequencing (ii) read-based reconstruction method: Amplicon sequencing. Due to a wide study on bacterial 16S rRNA and fungal ITS amplicon sequencing and whole sample sequencing by shotgun metagenomics, bioinformatics algorithms and tools are specially designed and developed. This plays a significant role in understanding diverse microbes inhabiting a varied environment.

#### Computational Approaches

Various approaches for reference and taxonomic profiling are implemented during sequence data analysis. In the case of OTU’s mapping for amplicon data, based on sequence similarity, three approaches are implemented, which are the following: (a) reference-based methods, which include mapping to reference databases like Greengenes and Silva database; (b) *de novo* OTU clustering method, which is for those data for which reference database match was very minimum or for those who rely on the *de novo* method (like UPARSE and UCLUST); (c) hybrid approach, which generally is initiated with reference mapping and unmapped sequences are further mapped by *de novo* clustering method, for instance, SortMeRNA (Edgar, 2013).

#### Databases

Various databases are developed and used as reference datasets during the taxonomic classification of the sequence data. The most common databases are Ribosomal Database Project, Greengenes, SILVA, and UNITE database (Pruesse et al., 2007; Cole et al., 2009; Abarenkov et al., 2010). Specific pipelines for the identification of any specific category of microbes are also developed. For instance, identification of specific fungal species from ITS data includes CloVR-ITS, Clotu, PIPITS, and Plutof (Abarenkov et al., 2010; White et al., 2013; Gweon et al., 2015).

#### Toolkits

Some of the frequently used bioinformatics toolkits in amplicon data analysis include QIIME (Caporaso et al., 2010), BioMaS (Fosso et al., 2015), and MOTHUR (Schloss et al., 2009). These toolkits facilitate multiple analyses at each step, starting from various kinds of sequence data preprocessing, like, denoising, merging of pair-end data, binning, demultiplexing, error correction, barcode-type analysis (Semenov, 2021) to diversity estimation. DADA2 algorithm is a recent pipeline for taxonomic identification, which uniquely produces amplicon sequence variant analysis rather than operational taxonomic units (OTU) (Callahan et al., 2016).

Workflow management systems available for genomic and metagenomic data where the users can apply the customized features and can build analysis and share data, including CAMERA (Seshadri et al., 2007), Galaxy (Giardine et al., 2005), and Ergatis (Orvis et al., 2010). Galaxy metagenomics toolkit contains six tools: fetching taxonomy, summarizing taxonomy, drawing phylogeny, finding of diagnostic hits, classification of diagnostic hits, and the Poisson two-sample test. Windshield splatter analysis is one of the first studies on metagenomic analysis using the NGS data analysis pipeline within the Galaxy workflow management system. Currently, there are many modular pipelines developed using such workflow systems.

There also exist stand-alone data analysis packages, developed using open-source tools by the Nanopore community, like Oxford Nanopore Technologies (ONT) cloud-based What’s In My Pot (WIMP) software, and MinION Detection Software (MINDS) for rapid species identification. Several bioinformatics applications have been developed implementing ONT’s framework for long reads’ analysis (Deshpande et al., 2019).

#### Tools

Computational tools and algorithms differ depending on the sequencing of data like amplicon-based or shotgun. In the case of shotgun data analysis, different software is used at different steps. Metagenome assembly is supported by the following algorithms starting from the traditional approach that includes overlap-based assembly and the most frequently used Kmer approach. Others include apriori algorithm, association rule mining algorithm, a binning algorithm like MaxBin 2, SPHINX, MetaCluster 5, sequence classification algorithm, and so on (Tandon et al., 2016; Saha et al., 2019). Classical metagenome gene predictors include Fgenesb and Critica/Glimmer which were evaluated by simulated data sets in a study by Kunin et al. (2008).

The shotgun based analysis is performed in various steps and tools used are the following: (i) assemblers, such as SOAP, Ray Meta, Snowball, MetaVelvet, MetaSPAdes, Meta-IDBA, MEGAHIT (Boisvert et al., 2012; Luo et al., 2012; Nurk et al., 2013; Li et al., 2015; Gregor et al., 2016); (ii) Binning of sequence fragments can be processed by taxator-tk, MaxBin2, Kraken, PhyloPytiaS+, Metaphlan, MEGAN and MetaBAT (Wood and Salzberg, 2014; Wu et al., 2014; Dröge et al., 2015; Kang et al., 2015; Gregor et al., 2016; Huson et al., 2016); (iii) metagenomics annotation and classification includes MetaGeneMark, PRODIGAL, KEGG, TIGRFAMs, COG, PFAM, eggNOG, and SEED (Overbeek et al., 2005; Zhu et al., 2010; Kanehisa et al., 2014; Powell et al., 2014; Garrido Oter, 2018).

Tools developed are even specific in mapping and characterization of organism type, like bacterial and archaeal annotations implemented by the functional annotation and gene calling pipeline, such as the command-line cg-pipeline and Ergatis web-based workflow management system. The electronic probe Diagnostic Nucleic acid Analysis (EDNA) tool utilizes NGS data for the detection of eukaryotic plant pathogens like fungi and oomycetes (Espindola et al., 2015). Similarly, virus-specific workflows are Viral MetaGenome Annotation Pipeline that was developed by using knowledge of specialized databases, such as mobile genetic elements collections and environmental metagenomes, to enhance the identification, classification, and functional analysis of viral gene products (Lorenzi et al., 2011).

Online servers for the study of metagenomics data uploaded by users can be comprehensively analyzed with MG-RAST, CAMERA, and IMG-M (Meyer et al., 2008). They also provide functional annotations and pathway reconstruction and facilitate the user for depositing, analyzing, sharing, both in public and private mode and visualization of the data in a single platform. All these resources help the researchers without access to high-performance computing facilities.

Comparative metagenomics, as its name indicates, is for multiple metagenome comparisons among different environments based on taxonomical and functional assignments in another important part of metagenome study. The first comparative metagenomics study introduced the knowledge of a gene-centric view of different environments (Tringe et al., 2005). Various studies on comparative metagenomics include Daphnia symbionts, modeling ecological drivers in marine viral communities, the role of microbes in human biology, poly-microbial black band disease of corals, biogas producing microbial communities, organic management, etc. (Meyer et al., 2017). This comparison platform will act as a tool in gaining a scientific understanding of microbial communities related to environmental perturbations. METAREP is one such tool that facilitates comparative metagenomics analysis along with statistical analysis like principal component analysis (PCA), non-metric multidimensional scaling (NMDS), and multivariate analysis^1^. Similarly, Integrated Microbial Genomes (IMG) system also serves as a comparative analysis resource facilitating the annotation and visualization of data^2^.

Metagenomics-wide association studies (MWAS) are a broader branch of metagenomics of recent interest. With its wider application, this study is successful but its use and implementation are still in its infancy stage because of various challenges. One such challenge focuses on the choice of tools, methods, and workflow (James et al., 2019). The study has also elaborated on the classification of tools in MWAS based on their analysis that includes phylogeny, non-phylogeny (statistical), hybrid tools (both statistical and phylogenetic methods), and machine-learning tools. Tools and software available for MWAS studies include the following:

(a) *Command-line software*: SEER (Lees et al., 2016), bugwas (Earle et al., 2016), Phenotype Seeker (Aun et al., 2018), PySEER (Lees et al., 2018), and HAWK (Rahman et al., 2018) and
(b) *Graphical user interface software*: Scoary (Brynildsrud et al., 2016) but also supports command-line function (Table 2).

**TABLE 2:** Software tools and algorithms in metagenomics and its association studies.

Functional category	Software/Tool	Sequencing approach data used/analysis	Area of study/Application	Web link
(1) Trimming	Trimmomatic	Illumina	All Omics	http://www.usadellab.org/cms/?page=trimmomatic
	FASTQC	All high throughput platform	All Omics	https://www.bioinformatics.babraham.ac.uk/projects/fastqc/
	PRINSEQ	All high throughput platform	Metagenomic	http://prinseq.sourceforge.net/
	SolexaQA	Illumina, Ion Torrent and 454 data	All Omics	http://solexaqa.sourceforge.net/
	FASTX-Toolkit	All high throughput platform	All Omics	http://hannonlab.cshl.edu/fastx_toolkit/
(2) *de novo* Assembly	RAY	All high throughput platform	Metagenomic	http://deNovoAssembler.sf.Net/
	MetaVelvet	All high throughput platform	Metagenomic	http://metavelvet.dna.bio.keio.ac.jp/
	Genovo	All high throughput platform	Metagenomic	https://pubmed.ncbi.nlm.nih.gov/21385045/
	CLC Genomics Workbench	All high throughput platform	All Omics	https://digitalinsights.qiagen.com/clc-genomics-workbench-features/
	Meta-IDBA	All high throughput platform	Metagenomic	https://i.cs.hku.hk/~alse/hkubrg/projects/metaidba/
	SOAPdenovo	Illumina	All Omics	https://github.com/aquaskyline/SOAPdenovo2
	Newbler	54 GS-series of pyrosequencing platforms	Genomic and Metagenomic	https://help.rc.ufl.edu/doc/Newbler
	ABySS	All high throughput platform	All Omics	http://www.bcgsc.ca/downloads/abyss/
	ALLPATHS-LG	Illumina	Genomic and Metagenomic	https://github.com/danforthcenter/bioinformatics/blob/master/docs/allpaths.md
(3) Reference-based Alignment	BWA	All high throughput platform	Short DNA sequence reads to a large reference genome	https://sourceforge.net/projects/bio-bwa/
	Bowtie	All high throughput platform	Short DNA sequence reads to a large reference genome	https://sourceforge.net/projects/bowtie-bio/
	MUMer	All high throughput platform	Short DNA sequence reads to a large reference genome	https://sourceforge.net/projects/mummer/
	BFAST	All high throughput platform	Short DNA sequence reads to a large reference genome	http://bfast.sourceforge.net
	MrFAST	Illumina	Short sequence reads	http://mrfast.sourceforge.net/pubs.html
	CloudBurst	All high throughput platform	Short DNA sequence reads to a large reference genome	https://sourceforge.net/p/cloudburst-bio/wiki/CloudBurst/
	SOAP	Illumina-Solexa	short oligonucleotides reads	https://academic.oup.com/bioinformatics/article/24/5/713/203564
	BLAST	All high throughput platform	Short DNA sequence reads to a large reference genome	https://blast.ncbi.nlm.nih.gov/Blast.cgi
	Novoalign	Illumina	Short sequence reads	http://www.novocraft.com/products/novoalign/
	MOSAIK	All high throughput platform	Short-Read Mapping	https://code.google.com/archive/p/mosaik-aligner/
(4) Annotation and comparison	MG-RAST	Taxonomic, Phylogenetic, functional and comparative analysis	Metagenomic	https://www.mg-rast.org/
	METAREP	Taxonomic, functional and comparative analysis	Metagenomic	https://www.jcvi.org/research/metarep
	DIYA	Functional analysis	Bacterial Genomics	https://sourceforge.net/projects/diyg/
	CloVR	Taxonomic and functional analysis	Metagenomic	http://clovr.org/
	RATT	Functional analysis	All Omics	http://ratt.sourceforge.net
	CAMERA	Taxonomic, Phylogenetic, functional and comparative analysis	Metagenomic	https://journals.plos.org/plosbiology/article?id=10.1371/journal.pbio.0050075#s4
	Eragatis	Functional analysis	All Omics	http://ergatis.sourceforge.net
	IMG-M	Taxonomic and comparative analysis	Metagenomic	http://img.jgi.doe.gov/m
	Blast against COG database	Functional analysis	All Omics	http://www.ncbi.nlm.nih.gov/COG/
	PICRUSt	Phylogenetic and functional analysis	Metagenomic	http://picrust.github.io/picrust/
	MEGAN	Taxonomic, Phylogenetic, functional and comparative analysis	Metagenomic	https://uni-tuebingen.de/fakultaeten/mathematisch-naturwissenschaftliche-fakultaet/fachbereiche/informatik/lehrstuehle/algorithms-in-bioinformatics/software/megan6/
	LEfSe	Taxonomic analysis	Metagenomic	https://huttenhower.sph.harvard.edu/lefse/
	DADA2	Taxonomic and phylogenetic analysis	Metagenomic	http://www.metagenomics.wiki/tools/16s/dada2
	QIIME	Taxonomic, Phylogenetic, functional and comparative analysis	Metagenomic	http://qiime.org/
(5) Statistical Analysis	STAMP	Statistical analysis of taxonomic and functional profiles	Genomic and Metagenomic	http://kiwi.cs.dal.ca/Software/STAMP
	PAST	Univariate and multivariate statistics, curve fitting, time-series analysis, data plotting, and simple phylogenetic analysis	All Omics, Paleontology and ecology	https://past.en.lo4d.com/windows
	FAST UniFrac	Diversity Analysis	Metagenomic	https://www.nature.com/articles/ismej200997
	R software Package	Statistical computing and graphics	All Omics	https://www.r-project.org/
	XLSTAT	Flexible Excel data analysis add-on for statistics	All Omics	https://www.xlstat.com/
	CANOCO	Multivariate data analysis and visualization	All Omics and ecology	http://www.canoco5.com/
(6) Metagenome Wide Association Studies	TreeWAS	Genome-wide and phylogenetic analysis	Metagenomic	https://github.com/caitiecollins/treeWAS
	bugwas	Genome-wide analysis	Bacterial Genomics	https://github.com/xiangzhou/GEMMA
	Phenotype Seeker	Genome-wide and phylogenetic analysis	Metagenomic	https://github.com/bioinfo-ut/PhenotypeSeeker/
	Scoary	Pan-genome association analysis	Metagenomic	https://github.com/AdmiralenOla/Scoary
	Magnamwar	Genome-wide analysis	Bacterial Genomics	https://cran.r-project.org/package=MAGNAMWAR
	PySEER	Pan-genome association analysis	Metagenomic	https://github.com/mgalardini/pyseer
	DBGWAS	Genome-wide analysis	Bacterial Genomics	https://gitlab.com/leoisl/dbgwas

## Metagenomics and Sustainable Agriculture

Agriculture challenges are the major concern in terms of the growing demand for food and bioenergy all over the world which has to be effectively addressed (Tilman et al., 2011). Environmental threats, including biodiversity loss, climatic change, water and land pollution, the low organic content of the soil, and degradation, have to be balanced accordingly. Many studies and strategies are planned in agricultural ecosystem biology but still need to be deciphered to gain deeper awareness and to meet the growing needs and negative impacts of the environment (Power, 2010). Soil fertility is a key player and depends on various factors, like, water availability and contamination, elements fluxes in soil like carbon, nitrogen, and phosphorus, and climatic conditions. But the most crucial is soil microorganisms’ dynamics. Under such cases, metagenomics will help in resolving challenges in agricultural sectors as it considers biotic and abiotic factors associated with it (Scholes and Scholes, 2013). Furthermore, the use and demand for biopesticides and biofertilizers in agriculture and advancement in sequencing and metagenomics analysis have led to the discovery of beneficial microbes for agriculture (Park et al., 2013).

Soil microbiomes are largely responsible for the biogeochemical cycles that support life on earth where they mediate the nitrogen cycle, carbon, and sulfur cycles, as well as in the transformation of oxidative and reductive states of metals like iron and mercury. They particularly influence the health of plants and their dependents by providing nutrients and vitamins, influencing developmental processes, and protecting the host from attack from virulence factors (Handelsman, 2003). All these show the positive phase of the microbial population with a balanced natural system. If this system is disrupted, it can lead to harmful effects like acid mine drainage, exposure of coal or copper mines to oxygen, and disruption of the earth leading to the production of high damaging waste. Most of these factors are currently addressed on a broader scale with metagenomics for instance soil microorganism dynamics is studied through diversity and functional analysis of soil microbial populations (Fierer et al., 2013).

**FIGURE 4:**
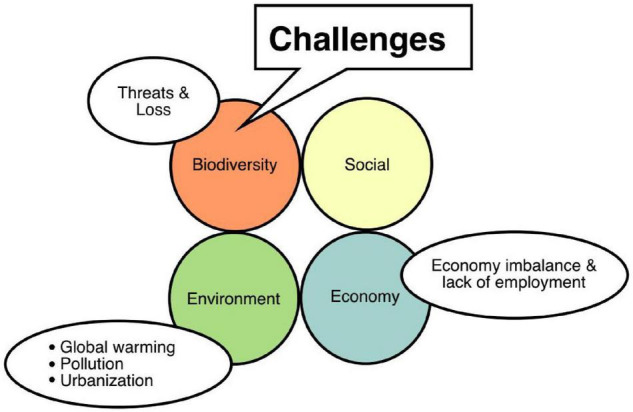
Threats to sustainable agriculture.

Rhizosphere harbors powerful microbiomes which play a significant role in maintaining plant health and productivity, nutrient cycling, and enhancing soil fertility. These microbiome complexities vary by plant species, soil type, host genotype, and land tillage system. They are the most complex ecosystem on earth and the rhizosphere microbiomes of various agricultural crops and their biocontrol characteristics have been studied (Subrahmanyam et al., 2020; Alawiye and Babalola, 2021). These studies and evidence reflect the importance of metagenomics application as a tool in unlocking the knowledge of biota that are influenced by abiotic factors in sustainable agriculture. Major challenges currently faced in agriculture in maintaining a balance between sustainability and increasing fiscal growth rate are the environmental threats, social threats, economic threats, and other factors like global warming, urbanization, and pollution (Gupta et al., 2018; Figure 4).

Soil metagenomics advancement stimulates new soil conceptions as “living” and “laboring” (Granjou and Phillips, 2019). It is also stated as a new agroecological revolution period with the use of biota for soil functioning. Overall soil metagenomics helps in reconfiguring biopolitical and bioeconomic relations and it may influence future (micro) biopolitical studies.

### Dimensions of Metagenomics Data in the Agricultural Ecosystem

The major perspective of the present review is to understand metagenomics applications in agriculture, including: (i) livestock health and disease, (ii) crop production and its environmental impact mainly abiotic stress, and (iii) design and development of novel products and their industrial applications. Among many such studies reported in the literature, we are presenting a few studies in Table 3 of this decade focused mainly on the agriculture sector.

**TABLE 3:** Overview of metagenomics applications in agriculture.

Agriculture samples used for study	Proposal/Hypothesis/Conclusion of the study using metagenomics approach	Country	Parameters evaluated	Method/Approaches used	References
Agriculture Soil and Rhizosphere	Proposed the functional metagenomics approach for mining novel biosurfactant for use in agrochemical industries.	India	Biosurfactant characterization	High throughput techniques: Functional Metagenomics	Sachdev and Cameotra, 2013
Roots of maize plants	Organic fertilizer shows positive feedback and can be a boost to sustainable agricultural practices. Novel endophytic bacteria groups also identified in maize for promoting growth and bio-industrial applications.	North-West University School Farm, Molelwane, Mafikeng, North West Province, South Africa	Maize roots microbiome cultivated from seeds grown in inorganic fertilization, and Organic fertilization and no fertilization soil	Shotgun sequencing	Fadiji et al., 2020
Sorghum cropped rhizosphere soil	Different fertilization managements including organic and chemical fertilization and its effect on soil microbial communities.	Battipaglia, Italy	Rhizospheric soil microbiome: Crop land (fertilized, chemical fertilized and compost amended) and grass land	Pyrosequencing	Lavecchia et al., 2015
Rhizosphere soil of barley and tomato	How iron nutrition, plant species and soil type shapes the rhizosphere microbiome?	Italy	Barley and tomato microbiome, characterized by different strategies for Iron acquisition.	454 pyrosequencing technology	Pii et al., 2016
Rice Root (*Oryza sativa* L.)	Endophytic microbial community profiling and its role such as nitrogen fixation in rice crop	India	Root endophytic microbial community of Indian rice (*O. sativa* L.)	Amplicon sequencing	Sengupta et al., 2017
South African uncultivated endemic plant species: *E. caput-medusae*, *Limeum africanum*, *Exomis microphylla*, and *Polygala garcinii*	Geometagenomics approaches: Viral diversity, host-pathogen interaction, Geminivirus case study that identified complex evolutionary dynamics of some of the highly divergent geminivirus species.	South Africa	Geminiviruses within two ecosystems containing both cultivated and uncultivated areas: Root microbiome	Geometagenomics-based approach	Claverie et al., 2018
Achatina fulica snails (agricultural pest): crop fluid	Comparative metagenome analysis of the first land snail crop microbiome from a highly invasive species, which has a wide-ranging diet and is capable of consuming different varieties of plants and substrates. The A. fulica holobiont represents a prosperous reservoir of novel GH genes and related modules, which will be of biotechnological application in eco-friendly biofuel production.	Rio de Janeiro, Brazil	Crop of an Invasive Snail: Metagenome	Shotgun sequencing	Cardoso et al., 2012
Plant, soil and compost samples of three leafy Asian greens – Brassica rapa var. parachinensis, Brassica oleracea var. alboglabra and Amaranthus spp.	Vegetable crop phytobiomes to understand the different functional aspects.	Lim Chu Kang, Singapore	Soil metagenome, leaf metagenome, root metagenome and compost metagenome	High throughput sequencing: HiSeq 2500 platform	Bandla et al., 2020
Wood tissue: grapevine plants	Two new viral species were identified including one belonging to Potyviridae family and one to the Bunyavirales order that are associated with grapevine plants.	Spresiano, Veneto region, Italy	Wood Metagenome	High throughput sequencing	Bertazzon et al., 2020
Tomato leaf samples	Population dynamics of begomoviruses associated with tomato crops, two novel Begomovirus species identified and potential viral adaptation to tolerance factor discovered.	Brazil	The metagenome of Neotropical Single-Stranded DNA Viruses	High-performance sequencing: HiSeq 2500	de Nazaré Almeida dos Reis et al., 2020
Wheat rhizospheric soil sample	How the diversity of the rhizosphere microbial community can have an impact on overall crop function?	Uttar Pradesh, India	Wheat Rhizosphere soil microbiome	Shotgun sequencing	Srivastava et al., 2020
Mummified peach fruits	Comprehensive overview of microbial communities in the mummified peach fruits using Metagenomics and Meta-transcriptomics approach.	Hoengseong, Gangwon province, South Korea	Mummified Peach Fruit comparative microbiome study	High throughput sequencing	Jo et al., 2020

Antibiotic-resistant genes in soil are majorly produced by the action of complex antibiotic-producing microbes which include aminoglycoside acetyltransferase, ADP-ribosyl transferase, ribosomal protection protein, aminoglycoside 6-adenyltransferase, transporters, and other antibiotic-resistant determinants and their resistant mechanisms are identified through functional metagenomics approach. The distribution of antibiotic-resistant genes in agricultural and non-agricultural metagenomic samples has been investigated and it was observed that a large percent of antibiotic resistance genes was produced by diverse bacterial communities in agricultural and gastrointestinal-associated metagenomes compared to marine and Antarctic samples (Durso et al., 2012).

Studies on the microbial establishment in the developing rumen are said to have implications in farm animals ecologically and physiologically that ultimately lead to productivity efficiency in mature animals. Metagenomic studies on fish gastrointestinal communities are focused on the identification of a healthy microbiome and its role in vertebrate health (Tarnecki et al., 2017).

### Applications of Metagenomics in Agriculture

#### Pathogen and Plant Disease Identification

Plant disease control, identification and characterization of the microbes, and prevention are successful and efficient with early detection of plant disease outbreaks and accurate diagnostic methods. Plant pathogens and pests have significant economic, ecological, and evolutionary consequences in agricultural ecosystems. Symptoms of attack of pathogens on plants are the major factors that help in diagnosing a disease by a plant pathologist. However, this is not a highly desirable method, as there is a gap existing in strain-level/species identification of the causative agent. For instance, in tomatoes “bacterial speck disease symptoms” are caused by different species of *Pseudomonas syringae* pathovar (pv.) tomato (Pto) (Cai et al., 2011). Similarly, “bacterial spot disease symptoms” on tomato leaves are caused by genus Xanthomonas but involve four different species (Jones et al., 2004). Another significant fact is that during diagnosis and crop rotation, different species of the same pathogen may have varied host ranges. In *P. syringae* pathovar (pv.) tomato (Pto), strain DC3000 could affect not only tomatoes but also leafy greens by existing in weeds of the Brassicaceae family (Yan et al., 2008).

Various methods are used in plant disease diagnosis for species identification, including gene sequence-based techniques with pure culture, 16S rRNA gene method but involve low resolution, whole-genome sequencing using pure culture, and antibody-based assays, such as ELISA (El Sheikha and Ray, 2014). However, limitations associated with these methods are a lengthy pathogen identification protocol to obtain pure culture and prior knowledge of pathogens. In the current scenario, all these limitations are overcome with metagenome sequencing and identification methods in agriculture.

Metagenomics in pathogen diagnosis has the potency to target multiple unique signature loci of pathogens in an infected plant. This approach has already been used to detect unknown pathogens in different organisms, including mammals, plants, and insects (Adams et al., 2009). Also, various farming practices influence the taxonomic diversity of the phyllosphere microbial community leading to unique signatures (Khoiri et al., 2021). Nevertheless, the protocol of this method should be targeted toward precise identification of causative agents as this involves DNA extraction, which may contain host sequences, other microbe sequences apart from pathogens of interest. Electronic-probe Diagnostic Nucleic acid Analysis or EDNA overcomes this with simulated metagenomes.

The bacterial tomato pathogen identification at the strain-level was achieved by metagenomics (Mechan Llontop et al., 2020). Fusarium wilt is a common vascular wilt fungal disease in many plants. In banana, a study with fusarium wilt infected fields was conducted by analyzing endophytic communities, which led to the recognition of bacterial communities having an impact on disease development, including Flavobacteriales (Kaushal et al., 2020). Simulated metagenome data analysis using EDNA was done for detecting fungal and oomycete plant pathogens (Espindola et al., 2015). Soil may also act as disease suppressive agent in agriculture by protecting the crops from harmful microbes. Mendes et al. (2011), with PhyloChip-based metagenomics, diagnosed key bacterial taxa and genes comprising 33,000 bacterial and archaeal species from soil to be involved in the suppression of fungal root pathogens (Mendes et al., 2011). This soil microbial consortia act as superorganisms in controlling and protecting plants and crops from harmful invading microbes.

Plant disease management can be made successful as in medicine through the transfer of microbiome by mixing disease suppressive soils with disease conducive soils (Gopal et al., 2013). Blackroot rot disease of tobacco in suppressive and conducive soil with rhizobacterial community study resulted in novel bacterial taxa that help as indicators of disease suppressiveness. Bacterial taxa with plant beneficial properties in disease suppressive soils were Pseudomonas, Azospirillum, Gluconacetobacter, Comamonas, Burkholderia, and Sphingomonadaceae, whereas Mycobacterium, Rhodobacteraceae, Bradyrhizobium, Rhodospirillum were more frequent in the conducive soil (Kyselková et al., 2009). Another global problem faced by potato growers annually is potato common scab caused by Streptomyces species. A similar study on suppressive and conducive soil identified various bacterial genera (predominantly Bacillus genus) in the disease-conducive soil (Rosenzweig et al., 2012).

#### Disease Resistance Breeding

Application and manipulation of plant-microbe interactions and microbe-microbe interactions with a strong scientific basis are being extensively used in agro-ecosystems. Their roles in improving plant health and productivity are a well-recognized in various studies. Microbial community engineering through plant breeding is another way of harnessing plant-microbe interactions (PMI). These interactions also provide insights in improving resistance breeding against diseases in crops, which is a promising approach in achieving sustainable agriculture (Rubiales et al., 2015).

Plant resistance breeding usually targets developing cultivars with the best performance over diversified environments. However, it is a very challenging task as holobiont varies in different environments. A study on lupine cultivars for resistance against *Fusarium avenaceum* in Canada was not successful, whereas it was found effective in Denmark and Germany (Chang et al., 2014). Therefore, it is also attributed to geographical location and environmental conditions. These studies show that selection under targeted environmental conditions will help in producing resistant cultivars. In grain legumes, PMI plays an important role in resistance breeding against root diseases (Wille et al., 2019). This is based on the hypothesis that plant functioning is mediated by plant metagenomes that encompass both internal and external microbes (Berg et al., 2017). Plants and plant metagenomes are comprehensively termed as holobiont necessary for plant-resistant breeding strategies (Wille et al., 2019).

Metagenomics application under the influence of resistance breeding in Phaseolus vulgaris (common beans) against fungal root pathogen Fusarium oxysporum (Fox) with rhizosphere microbiomes was conducted. As rhizosphere microbiomes are involved as the first line of defense against root infections and diseases, the use of metagenomics in rhizosphere engineering has also been studied (Hakim et al., 2022). This study identified microbes, including Pseudomonadaceae, bacillaceae, solibacteraceae, and cytophagaceae, to be predominant in resistant cultivars of common beans. Metagenome analyses further explained that unique functional traits like protein secretion systems and biosynthesis genes of antifungal phenazines and rhamnolipids were dominant in the rhizobacterial community in Fox-resistant cultivars (Mendes et al., 2018).

In another study, comparative analyses of rhizosphere metagenomes from resistant tomato variety, Hawaii 7996 and susceptible variety, Moneymaker to soil-borne pathogen *Ralstonia solanacearum* revealed the abundant presence of flavobacterium genome in resistant variety, following which rhizosphere microbiota from resistant plants when transplanted suppressed disease symptoms in susceptible plants (Kwak et al., 2018). All these pieces of evidence prove the impact of metagenomics in resistance crop breeding.

#### Plant Pest Control

Microbes act as prominent biocontrol agents in the following ways: inhibition of pathogenic genes by impairing the pathogens with quorum sensing, production of antagonistic molecules in plant tissues, influence on plant defense mechanism through hormones production. There are also microbes that support pathogens through their mutual relationship like the Enterobacteriaceae family (Erlacher et al., 2014). So in the biocontrol mechanism microbes having negative benefits on pathogens can be used, on the contrary, microbes showing positive effects with pathogens can be targeted to break the symbiotic relationship in plant protection methods (Berg et al., 2017).

Plant microbiota act as substitutes to environmentally unfriendly pesticides and agrochemicals. It also mediates significant functions, including fitness, nutrient availability, pathogen or pest control, and stress tolerance. Microbial population manipulation in the rhizosphere is directly associated with crop health and productivity of crops. This microbial population is studied extensively using metagenomics as a prime tool. These strategies studied with metagenomics will be helpful for the control of plant diseases (Parakhia and Golakiya, 2018). Plant-associated metagenomes help in recognizing a large number of signatures involved in these interactions, but the traits produced through these interactions are yet to be extensively studied. Metagenomics the emerging omics technology shows that analysis of the microbiome and its function may provide insights and a paradigm shift in deciphering the holobiont functioning related to health and associated biocontrol measures.

The recent study by Poudel et al. (2019) on tomatoes with a metagenomics approach observed rootstock genotypes on endosphere and rhizosphere microbiomes which have the potential to select a candidate for the biocontrol process (Poudel et al., 2019). Organic crops are potentially enriched with their own biocontrol agents. Schmid et al. (2011) study on the grapevine-associated microbiome in organic and conventional fields evidenced the role of a biocontrol agent Aureobasidium pullulans, as well as the copper-detoxifying fungus in the organic field (Schmid et al., 2011).

Metagenomics study on disease and surviving plant microbiota for future plant health demonstrated that surviving plant microbiomes are linked with specific rare taxa having pathogen-suppressive tendencies or else involved in encoding antimicrobial compounds, these taxa included bacterial microbes like Pseudomonas and Bacillus (Wei et al., 2019). Fusarium Head Blight (FHB) is a devastating global disease caused by *Fusarium graminearum, a fungus* affecting small grain cereals. It is devastating, as it poses threat related to health diseases by producing mycotoxins which affect yield and seed germination. Therefore, a project using the metagenomics approach is proposed to interpret the wheat head microbiome and its dynamics during FHB disease development^3^. It also has the potential to reveal growth-promoting microbes or new pathogen antagonists, thereby aiding in the identification of novel biocontrol promoters.

Pest management strategies also involve microbial and viral enzymes like chitinases for use as bio-pesticides and have promising fungicidal, nematicidal, and insecticidal activities. Unraveling novel chitinases for use as a biocontrol is possible with a powerful tool, such as metagenomics. Metagenome-sourced chitinases have been studied in terrestrial and aquatic ecosystems with their distribution varying among abiotic factors. One such study is the identification of Chi18H8b, a novel chitinase with antifungal activity against *Fusarium graminearum* and *Rhizoctonia solani* (Berini et al., 2017). These novel metagenome-sourced chitinases were extracted from wastewater contaminated soil, chitin-enriched soil samples, pig feces, and sediments (Cretoiu et al., 2015). Metagenomics contribution in biocontrol agent identification is promising and expected to show greater application in near future.

#### Weed Management

With the global increase of herbicide-resistant weed communities, there is a need for a strategy targeting the different mechanisms of action. Development of metagenomics application for natural product discovery paves way for approach in weed management research which leads to innovative control strategies in cropping and farming systems. This approach based on high-throughput technology can accelerate the rate and scale of herbicide development in the agriculture and pest management sectors.

Transformation of traditional methods with metagenomics techniques in the discovery of novel and natural products like enzymes and antibiotics from microbes having antimicrobial and phytotoxic effects are increasing (Dhanjal et al., 2020b; Wani et al., 2022). Metagenomics-based functional screens offer a natural drug discovery potential for the segregation of weed suppressive compounds from microbes that targets the wider plant range. The recent focus on bioherbicide research is targeting major crops and turfgrass landscapes.

Metagenomics-based functional screens in weed management involve two approaches (i) novel herbicide isolation and (ii) herbicide resistance genes identification. Lucas (2011), Genetically modified crops resistant to herbicides through recognition of herbicide resistance genes from soil microbes were studied (Lucas, 2011). Another study was conducted, leading to the discovery of glyphosate resistance and glyphosate degrading abilities using microbes (Staub et al., 2012). It is estimated that the finding of a novel antibiotic in actinomycetes may involve hundreds of clone screening, but metagenomics along with robotics techniques may speed up the acceleration process. A similar study has been done on cellulase with hundreds of clone libraries (Mewis et al., 2011).

Although, the number of studies linking metagenomics in weed management is limited there are many promising studies conducted to show its potential applications. For example, application of metagenomics and metatranscriptomics approaches in studying seed microbiome population and function for identification of novel bioherbicides (Müller-Stöver et al., 2016) and use of metagenomics functional screening approach for microbiome selection on small plants, such as duckweed (Lemna minor L.), algae or leaf spot assays, to test success rate, following a large-scale screening in the greenhouse (Kao-Kniffin et al., 2013). Metagenomics along with the metabolomics approach helps in the identification of novel herbicide compound discovery. Numerous antibiotics were obtained from Streptomyces spp. using the metagenomics approach have the ability to suppress the weed. The role of new sequencing technologies for weed establishment and weed prevention along with the exploitation of the knowledge in search of new biocontrol agents against weeds based on soil and plant microbial communities is well reported (Trognitz et al., 2016).

#### Abiotic Stress Management

Microbes associated with plants/crops, including soil microbes and rhizosphere microbiomes, contribute to a wide range of functions that are needed for plant productivity, like mineralization of soil organic matter, nutrient cycling, stimulating disease resistance, and responding to abiotic stresses like drought, salinity, pollution, etc. There is extensive information about the ability of specific soil microbes to influence abiotic stress tolerance in plants, for example, the impact of rhizosphere microbiome on some plant species survival under extreme stress conditions (Jorquera et al., 2012).

Abiotic stress players are non-living components but pose a great threat and unfavorable conditions and challenges in agriculture. Plant response depends on the type of stress like extreme temperature, drought, salinity, waterlogging, soil pollutants, hazardous compounds, such as heavy metals, and the extent to which the condition is affecting the plant. They are the foremost limiting factors in crop productivity. Crop plants need to cope with these adverse external pressures of the environmental conditions with their innate biological mechanisms, failing which plant growth, development, and productivity suffer (Meena et al., 2017).

To perceive the cumulative role of these multiple interactions in plants along with its ability in abiotic stress management (ASM), a broader scale study, such as metagenomics, is needed. Zolla et al. (2013), work on drought stress conditions in Arabidopsis thaliana using the pyrosequencing approach reported the robust set of soil microbes having an ability to sense abiotic stress and increase plant biomass production along with reduced expression of drought response marker genes (Zolla et al., 2013). A similar study on wheat rhizosphere for abiotic stress management reported the wider and diversified role of metagenomics in ASM where metagenomics of wheat along with its application in climate-resilient genotypes development is elaborated (Ahlawat et al., 2018).

Soil pollution is another important abiotic feature associated with plant damage and disease susceptible environments. Response to polycyclic aromatic hydrocarbons pollution using 16S rRNA gene pyrosequencing approach was studied on Trifolium (Kawasaki et al., 2012). Pollutants like detergents, oxidants, and glucose also pose severe losses to agriculturists. The promising use of functional metagenomics in unrevealing proteins that are tolerant to such pollutants from agricultural soil is well reported. Alkaline β-glucosidases enzymes showing high resistance toward severe detergents, glucose, and oxidants are identified by this technique.

In another study on proteins like ACC-deaminase, genes involved in stress alleviation, using metagenomics libraries reported the functioning of *acdS* operon from uncultivated endophyte in potato (Nikolic et al., 2011). Salt-tolerant genes from pond water metagenomics libraries can be used in developing salt-tolerant recombinant microbes and transgenic plants. Comparable study on acid mine drainage led to the identification of genes that tolerate low temperatures like cold-shock proteins, anti-freeze protein, pH homeostasis, and compatible solutes production pathways using the metagenomics approach (Liljeqvist et al., 2015). Functions involved in improving plant stress resistance activity like detoxification of ROS and quorum sensing can also be identified through the metagenomics approach with endophytic bacterial residents of rice roots (Sessitsch et al., 2012).

A novel approach using endophytic bacteria to alleviate various stress conditions is gaining significance in agriculture. Kunda et al. (2018) study on bacterial endophytes from roots of rice plants grown in the coastal saline zone identified the microbial genera with plant growth-promoting potentials in a high salt environment and also proposed the use of these microbial genera for designing cultivation strategies in saline conditions (Kunda et al., 2018). Methe et al. (2017) study on functional genes that differentiate maize phyllosphere metagenomes in two distinct conditions: drought and well-watered identified unique taxa involved with potential growth-promoting traits (Methe et al., 2017).

#### Post-harvest Management

Post-harvest loss is one of the major concerns worldwide. It is a wider sector, covering a long chain of processes from production in a field to food in consumer plates (Buchholz et al., 2018). It includes several steps, such as harvesting, protection, preservation, preparing, packaging, transportation, and marketing. It is stated by the Food and Agriculture Organization (FAO) that ‘Hunger is still one of the most demanding challenges, still the agriculture throughout the world is producing more than enough food.’, but a huge amount of food produced gets lost on its way from the field to consumer. It is also estimated by FAO that about 15--50% of food produced in developing countries is lost after harvest due to various abiotic factors like drought, high temperature, huge rainfall, the wrong procedure of harvesting, physical damage, or contamination by microbes^4^.

Post-harvest food loss is defined, as measurable qualitative and quantitative food loss along the food process chain, beginning from the time of harvest until its consumption. Food losses are attributed to loss during harvest, transport, loss in quality due to undesired microbial growth or pathogen attack, rancidity, water loss, etc. (El Sheikha and Xu, 2018). Post-harvest loss in potato production in Switzerland showed that about 50–55% losses are due to pathogen attack, water loss, saccharification, etc. (Willersinn et al., 2015). However, plant microbiota dynamics have a symbolic role in post-harvest food loss by influencing the storage of crops. However, many studies on plant microbiota are focused on crop productivity, but still, the plant microbiome’s role and impact on processing, packaging, storing, and marketing is mostly unexplored.

Pathogen-induced post-harvest crop loss caused by microbial activity, including bacteria and molds, is obvious. Plant microbiomes consist of complex microbial interactions of potentially mutualistic, commensal, and pathogenic microbes colonizing as in crop plants. The dynamics and role of the bacterial community of potato tubers during infection with soft rot pathogen named *Pectobacterium atrosepticum* was studied (Kõiv et al., 2015). The soil microbial communities on flowering phenology and reproductive fitness of Boechera stricta, a wild relative of Arabidopsis were tested and it was concluded that the soil microbiome is the possible driver contributing at flowering time, to differential selection observed between habitats through the metagenomics approach (Wagner et al., 2014).

The *Vitis vinifera* L. cv. Corvina grapes are popular for the production of unique wines, like Amaron. This unique feature is strongly linked to the post-harvest grape withering process. With the use of whole metagenome sequencing, insights into the microbiome of Corvina withered berries revealed pertinent variations attributable to post-harvest withering conditions. This knowledge and management of the withering process in Corvina will have an impact on winemaking (Salvetti et al., 2016). Meta-omics technologies involving meta-genomics, meta-transcriptomics, meta-proteomics, and meta-metabolomics are at the earlier stage in post-harvest studies. This *omics* approach will certainly bring revolution in our understanding and knowledge of post-harvest biocontrol systems, post-harvest physiology, and foodborne pathogens (El Sheikha et al., 2018).

An important feature that has to be noted is microflora associated with different parts of a fruit/vegetable should be considered when preparing strategies for biocontrol systems involving post-harvest disease management. A work by Abdelfattah et al. (2016) reported the diversity in fungal microflora among various parts of harvested apples and as well as in farming techniques like organic or conventional methods (Abdelfattah et al., 2016). Mazzola and Freilich (2017) also emphasized the need for meta-omic studies to understand the potential and functional aspects of the microbial community in post-harvest management strategy (Mazzola and Freilich, 2017).

#### Discoveries From Metagenomics in Agriculture

Metagenomics as a tool resulted in many discoveries. First, bacteriorhodopsin was discovered due to metagenomics as a novel gene product. Discoveries on microbial rhodopsin through functional metagenomics studies were stated (Pushkarev et al., 2018). Heliorhodopsins, which include diverse members of the rhodopsin family, are detected from microorganisms distributed globally. Further, novel biomolecules with antimicrobial activity and new proteins of known families like DNA polymerase and RecA were also identified. Microbial communities are agents and insight providers on biogeochemical cycling, energy and nutrient cycling, and metabolic potential. Multi-metagenome complete genome recovery from metagenome has led to link genomic analysis, which includes observations on gene function, genome structure, lateral gene transfers, and population genetics.

Metagenomics is probably used as a tool in the pathogen detection of agriculture. Synthetic metagenomics involves the identification of novel genes through mining sequence databases and metagenomics data for the gene of interest, followed by its chemical synthesis. This approach has led to the identification of the enzyme, methyl halide transferase, which is prominently used in biofuel production in the agriculture industry (Culligan et al., 2014). Biosurfactants have been identified through metagenomics studies, which are used as emulsifiers and detergents in various sectors, including pharmaceutical, agricultural and ecological functions (Thies et al., 2016).

#### Source of Novel Molecules/Compounds

The metagenomics approach has led the way in novel discoveries of genes, enzymes, and antibiotics. Functional metagenomics will accelerate the rate of discovery of new molecules from the microbiome community and it paves ways to investigate natural products from uncultured microbes. Metagenomics has been used as routine tools for novel natural product discovery during larger-scale exploration of bacteria coupled with genome engineering of naturally privileged heterologous expression hosts under larger-insert environmental DNA cloning methods (Iqbal et al., 2016). These natural products, which were discovered through the metagenomics approach have a number of characteristics, such as antimicrobial, hydrolytic, and phytotoxic compounds, and are being used in industry, agriculture, and biomedicine fields.

In agriculture, these novel products are antibiotics and have the potential to suppress weeds, act as biocontrol agents by plant-growth promotion, pathogen suppression, and plant disease resistance as well as effective pesticides. For example, buffalo rumen is used to isolate enzymes with unique features that can survive in extreme temperatures or pH (Singh et al., 2012). In another examination, novel hydrolase diversity was retrieved from bovine rumen microflora with a metagenome library and had a role in biocatalysis. Similarly, novel endo-α-1,5-L-Arabinanase was discovered from cow rumen with an endo-acting mechanism with arabinotriose as the final product (Wong et al., 2009).

Agricultural soil is also a rich source of novel compounds with diverse applications in industry. Novel phytase genes were identified from an agricultural soil microbial community for identification of phytase activity in a metagenomic library (Tan et al., 2014). In a different survey, a novel salt-tolerant, chitobiosidase was derived from chitin-amended disease-suppressive agricultural soil with the use of a metagenomic library (Cretoiu et al., 2015). Rhizospheric soil samples collected from three different *Solanum phureja* farms located in the Cundinamarca Andean Plateau, Colombia revealed unique lipase, esterase, and protease enzymes that have a biotechnological application (Calderon et al., 2019).

#### Source of Biosurfactants

Another promising role of functional metagenomics is in green biosurfactants analysis from uncultured microbes in the agriculture industry. It has the potential to replace harsh chemical surfactants. In biosurfactants synthesized by the rhizosphere, plant-associated microbes play a vital role and have a wider application in industries, including agriculture. They are responsible for biofilm formation, motility, signaling process, plant-pathogen destruction, the bioavailability of nutrients, agricultural soil quality enhancement through soil remediation, plant growth promotion, etc. There are different types of biosurfactants obtained from bacteria, yeast, and fungi based on their physiochemical properties, such as lipopeptides, glycolipids, phospholipids, neutral lipids, polymeric, and fatty acids. In the bioremediation process, a biosurfactant is employed for the effective removal of hydrocarbon and metal pollutants that are tightly bound to soil (Pacwa-Płociniczak et al., 2011; Sharma et al., 2021) for enhancing the degradation of certain chemical insecticides in agricultural soil. The first report of biosurfactant identification was by using the metagenomic library from environmental DNA that is N-acyltyrosines with N-myristoyltyrosine (Thies et al., 2016). The application of metagenomics in deciphering the novel biosurfactants from the marine environment is also reported (Gudiña et al., 2016). Moreover, N-acyltyrosines exhibited antibiotic activity across varied bacteria.

#### Source of Natural Product

Different industries depend on various agricultural products in their application. Here, products discovered using a metagenomics approach from agriculture-related fields to use in industries are discussed. In second-generation biofuel production, the focus is on the use of sustainable and alternative energy management. In this case, the industrially reasonable and economic conversion process involves the use and conversion of lignocellulosic biomass into biofuel molecules with cellulolytic enzymes. Tiwari et al. (2018), elaborated a study on deciphering the novel and effective cellulases from different environmental niches by uncultivable metagenomic approaches for their strong utilization in bio-refineries (Tiwari et al., 2018). Agricultural production-scale biogas plants provided new insights into microbial community composition and the genetic potential of important communities involved in biogas production (Bremges et al., 2015). Hemicellulolytic enzymes used in biofuel production are obtained from ruminal metagenomic libraries (Duque et al., 2018). Asymmetric synthesis of chiral alcohols using microbes is also studied (Saravanan and Suneetha, 2017). Table 4 describes the novel products detected using metagenomics approaches.

**TABLE 4:** List of natural products discovered using a metagenomics approach.

S. No.	Novel compounds	Source	References with PubMed ID/Cross-link
1	Lactocillin	*Lactobacillus gasseri*	Donia et al., 2014, 25215495
2	Lugdunin	*Staphylococcus lugdunensis*	Zipperer et al., 2016, 27466123
3	4-hydroxybutyrate dehydrogenase	Soil	Henne et al., 1999; Henne et al., 1999, 3393
4	Alcohol oxidoreductase	Field Soil	Knietsch et al., 2003, 12620823
5	Alpha amylase	Cow dung	Pooja et al., 2015, https://link.springer.com/article/10.1007/s12088-014-0487-3
6	Amidase	Soil	Gabor, 2004, http://irs.ub.rug.nl/ppn/265777593?pFullItemRecord=ON
7	Amylase	Soil	Gabor, 2004, http://irs.ub.rug.nl/ppn/265777593?pFullItemRecord=ON
8	Asparaginase	Soil	Arjun et al., 2018, 29127643
9	Beta-galactosidase	Infant feces	Xin et al., 2019, 31511933
10	Biotin production	Soil	Entcheva et al., 2001, 11133432
11	Cellulase	Sediment enrichment, Buffalo rumen	Rees et al., 2003, 12845554; Shah et al., 2017, 28733938
12	Chitin synthase	*Tribolium castaneum strain GA-2*	Arakane et al., 2004, 14871625
13	Chitinase	Soil characterized as suppressive to club root disease of cabbage	Hjort et al., 2014, 24121932
14	Endosulfan degrading protein	*Mycobacterium sp. strain ESD*	Sutherland et al., 2002, 12450848
15	Erdacin	Desert Soil	King et al., 2009, 19621341
16	Exosialidase	Freshwater thermal hot spring	Chuzel et al., 2018, 30249617
17	Extradiodioxygenases	Activated sludge	Suenaga et al., 2007, 17686025
18	Fasamycins A, B	Soil	Feng et al., 2012, 22224500
19	Fatty acid enol esters	Soil	Brady et al., 2002, 12188643
20	Glycopeptide- and lipopeptide-like antibiotics	Soil	Owen et al., 2013, 23824289
21	Hemicellulase	Degrading wheat straw	Maruthamuthu et al., 2016, 26822785
22	Indirubin	Soil	MacNeil et al., 2001, 11321587
23	Isocyanide derivatives of tryptophan	Soil	Brady et al., 2004, 16206308
24	Lignocellulose degrading enzymes	Porcupine microbiome	Thornbury et al., 2019, 30601862
25	Methyl halide transferase	Biomass	Bayer et al., 2009, 19378995
26	N-acyl tyrosine	Soil	Brady and Clardy, 2000, https://pubs.acs.org/doi/abs/10.1021/ja002990u
27	N-acyl-amino acid synthase	Soil	Brady et al., 2002, 12188643
28	Nitroreductases	pharmaceutical industry effluent	Sree et al., 2019, https://www.sciencedirect.com/science/article/pii/S0964830519305803
29	Novel Biindole Pigment	Marine Sponge Halichondria okadai	Abe et al., 2012, https://www.journal.csj.jp/doi/abs/10.1246/cl.2012.728
30	Novel biocatalysts	Field Soil	Voget et al., 2003, 14532085
31	Novel Isoprene-Degrading Proteobacteria	Soil and Leaves	Larke-Mejia et al., 2019, 31866954
32	Palmitoylputrescine	Bromeliad tank water	Brady and Clardy, 2000, 15528554
33	Polyketide syntase	Soil	Ginolhac et al., 2004, 15345440
34	Terragine A thru E	Soil	Wang et al., 2000, 10956506
35	Transaminase	Environmental DNA of uncultivable archaea and bacteria	Pawar et al., 2018, https://pubs.rsc.org/en/content/articlehtml/2018/ra/c8ra02764a
36	Turbomycin A, B	Soil	Gillespie et al., 2002, 12200279
37	Violacein and deoxyviolacin	Soil	Brady et al., 2001, 11418029
38	β-galactosidase	Soil	Liu et al., 2019, 30529535
39	β-Lactamase	Soil	Gabor, 2004, http://irs.ub.rug.nl/ppn/265777593?pFullItemRecord=ON
40	β-N-acetyl hexasoaminidase tri-functional enzyme	Oil spilled mangroves	Soares et al., 2017, 28952541

Other industries include pharmacy, which involves antibiotics and pharmaceuticals identified from agricultural soil. Antibiotics discovered are indirubin, deoxyviolacein and violacein, N-acyltyrosine, terragine (mycobacterium-inhibiting antibiotic), acyltyrosines, and turbomucin A and B.

#### Identification of Biosynthetic Molecules

Functional metagenomics has led to the detection of carotenoids and metatricycloene, a biosynthetically and structurally significant polyene. Metatricycloene is the first biosynthetically complex natural product to be obtained from *Streptomyces* species by metagenomics screening methods. Large numbers of herbicidal compounds have been isolated from soil bacteria which paved the way for the development of popular herbicides, like glufosinate (Kao-Kniffin et al., 2013).

Biosynthetic genes and proteins associated with insecticidal activity are discovered through this approach. For instance, insecticidal active proteins were isolated from Xenorhabdus nematophilus, and discovery of pyripyroprene an insecticide from Penicillium coprobium PF1169 (Hu et al., 2011). Pest management strategies, which were discussed in this review, explain the application and design of microbial-derived management strategies in agriculture. A study on Serratia entomophila strain Mor4.1, a bacterial pathogen of several soil pests from genera: Phyllophaga and Anomala lead to the discovery of cell membrane protein having a toxic effect, which is proposed to be used in designing biocontrol project (Rodríguez-Segura et al., 2012).

#### Use in Genetically Modified Crops

Metagenomics application for genetically modified crops is through identification of herbicide resistance genes from soil microbes, which can be used in engineering genetically modified crops. The best example is the discovery of glyphosate resistance and glyphosate degrading abilities from microorganisms (Staub et al., 2012).

#### Antibiotic-Resistant Genes

Metagenomics analyses play important role in the identification of antibiotics resistant genes in agriculture. This antibiotic resistance gene from agricultural soil contaminates groundwater. In addition, the clinical consequences caused by it will have an adverse effect, if, consumed by humans and animals. Antibiotics resistance genes of about 9 aminoglycosides and one tetracycline were identified from soil (Riesenfeld et al., 2004).

## Metagenome-Wide Association Studies

### Predictive Biology of Crops

Numerous studies on metagenomics in agriculture have led to the association of microbial communities in crop growth factors like crop productivity, growth rate decline, etc. Recent works of research in agricultural metagenomics are being focused on Metagenomics Wide Association Studies (MWAS). MGWAS and Genome-Wide Association Studies (GWAS) have striking similarities between challenges and pitfalls and elaborated on three key points in MWAS analysis (Weissbrod et al., 2018). These are (i) uniform data format adoption (ii) stringent statistical criteria and (iii) treating microbiome and host genome as specific entities. It aims at identifying genetic variants in microbial genomes that are associated with host variation in microbe phenotypes, like genetic variation affecting phenotypes, including carriage (in humans) and virulence in microbes.

In the MWAS approach, the microbial community identified through the metagenomics approach is used as a tool for analysis along with its abundance. The abundance of taxa is used as an explanatory variable for MWAS studies. MWAS is used to study many host-microbe interactions as a way to uncover microbial communities that are important for host health. A study by Crespo-Piazuelo et al. (2019), suggested that about 39 candidate genes may be involved in regulating microbiota composition, as well as responsible for the association between host genome and gut microbiota in pigs through MWAS (Crespo-Piazuelo et al., 2019). It is used in defining Enterohemorrhagic Escherichia coli Colonization. Further, it has been found that the plant loci responsible for defense and cell wall integrity affect microbial community variation in Arabidopsis thaliana (Horton et al., 2014).

Metagenomics-wide association studies has also been applied to regulate genes that are important for species-specific phenotypes in Helicobacter pylori, human diseases like asthma, obesity, and diabetes (Dutilh et al., 2013; Karlsson et al., 2013). Very few MWAS studies have been done in an agricultural context. Some of these are on crops like soybeans, millets, grapes, and strawberries (Debenport et al., 2015; Chang et al., 2017). In these studies, the linking of MWAS based on taxon abundance and crop productivity rates are emphasized, which is elaborately discussed in the following sections.

Theories, such as neutral (stochastic) and niche (deterministic), are subject to managing ecological populations. Rhizosphere microbiome is regulated by a deterministic approach, while, habitats in bulk soil are dictated by stochastic theory. Niche mechanisms depend on local habitat circumstances and their niche requirements. Further, the shaping of niches and helping of microbial community assembly is through physical-chemical soil characteristics and products derived from plant roots. A total of 25 abiotic soil characteristics, including latitude and longitude of sampling places, clay, silt, sand, and 12 types of elements percentage, pH, organic matter, cation exchange capacity along with percent saturation of elements and their influence on agricultural crops, were studied (Chang et al., 2017).

Genetic variation in microbes is altogether different from human genetics. The three major variations are (i) single nucleotide polymorphisms (SNPs) and insertion-deletion mutations (INDELs), (ii) gene presence and absence, and (iii) bi-allelic SNP variation that is responsible for the phenotype of interest. Understanding the type of variation is a fundamental key in selecting tools and methods in MWAS analysis. Various tools used for the MWAS study are elaborated in detail in the tools and approaches section.

### Application of Metagenomics-Wide Association Studies

#### Crop Productivity Rates

Keen interest in crop productivity linked to MWAS is exponentially increasing, studying the differences in crop productivity rates at a location within a field, despite having the same features related to crop genotype, management practices, and cropping history. Assumptions behind this crop productivity differences are attributed to unequal distribution of bulk soil biotic and abiotic factors in a location within agricultural fields.

With regard to crop productivity, it is a quantitative trait influenced by several factors, which includes physical and chemical analysis for abiotic factors and the next-generation metagenomics sequencing approach in relation to biotic factors. There are several abiotic features but few that significantly impact crop productivity, including water, weather conditions like temperature, rainfall, and nitrogen availability. Further, soil characteristics that are much important like soil moisture, soil physical compaction, and drainage differences within agronomic fields should also be considered.

Limiting factors of productivity in relation to biotic stress are (i) yield performance, which is affected by pest, pathogen and influenced by root nodulation (ii) genotype of crop variety includes genetics related to the following features: water and nitrogen utilization efficiency, photosynthesis, and productivity performance, disease, and pest resistance. de Almeida Lopes et al. (2016), described the influence of crop genetic features on rhizosphere microbiome structuring (de Almeida Lopes et al., 2016).

In addition, plant growth-promoting rhizobacteria (PGPR) are playing a key role in crop productivity leading to high crop production and suppressing the development of diseases. Microbiome structure in the phyllosphere and rhizosphere differ between plant species, and also with species genotype. Peiffer et al. (2013), studied the plant-microbe interactions through genome wide-associations which can be integrated into plant breeding (Peiffer et al., 2013).

#### Intercropping Analysis

Intercropping systems are known to positively affect crop growth and yield by potentially recruiting favorable microorganisms at the root zone of a crop. The impact of intercropping on the structure of microbiomes is widely studied by methods from terminal restriction fragment length polymorphism, denaturing gradient gel electrophoresis, phospholipid fatty acid analysis to high throughput sequence-based approaches. Intercropped soil samples showed frequently large-magnitude differences in the abundance of beneficial microorganisms in crops.

Applications of intercropping strategy have led to stimulation of microbial ecology that increased soil enzyme activity and resulted in high yield (Debenport et al., 2015). Benefits of intercropping include improvement in nutrient content, soil moisture profiles, microfauna of soil, soil enzyme activity, phospholipid fatty acid biomarkers, root zone microbiome, increase in crop production, soil organic carbon, etc. Soil organic matter plays a very potent role in agriculture and its biodegradability depends on compounds like organic and humic acids, proteins, and lignin along with pH and oxygen level, including microbial quality, composition, size, and activity.

Microorganisms’ spatial and temporal interactions with organic matter dictate the overall microbial population at a specific site. The increase in soil organic content depends on soil type. For instance, in semi-arid soils, it influences the arbuscular mycorrhizal diversity, whereas, in polar desert soil as well as in medium and low-level saline soils, bacterial communities are dominated (Van Horn et al., 2014).

#### Agronomic Field: Rotation With More Crops

Rotation is a cultural practice in agriculture to restrict soil-borne pathogens causing diseases. It has been observed that the same cropping pattern over a long period of time leads to reduced yield potential. Examples are apples, almonds, strawberries (Mazzola and Manici, 2012). The soil-borne pathogen, which affects a large number of plant species usually, penetrates through the roots from infected soil or damages the host roots, making them susceptible to infection. An example includes fungal pathogens *Verticillium dahliae* Kleb, *Rhizoctonia* sp., *Cylindrocarpon destructans*, *Fusarium oxysporum*, *Fusarium solani*, *Pestalotia longiseta*, *Pythium* spp., *Aphanomyces euteiches*, *Macrophomina phaseolina*, Sclerotium and nematodes, such as *Pratylenchus penetrans*, *Meloidogyne hapla*, and *Globodera pallida*.

Recent advances in agriculture are using biofumigation and anaerobic soil disinfestation (ASD) methods in controlling these pathogens and pests. Biofumigation is preferred over chemical methods of fumigation involving methyl bromide and chloropicrin. Biofumigation is used for brassica species that generate isothiocyanate as biologically active green manures. Crop rotation with non-host crops resulted in decreased incidence of infections and an increase in crop production. For example, Neubauer et al. (2014), assessed systematically the use of brassicaceous green manures to Verticillium dahlia but the results concluded that their efficiency in controlling the pathogen depends on soil organic conditions (Neubauer et al., 2014). In another study, wilt occurrence in strawberries is reduced by broccoli or lettuce crop rotation (Njoroge et al., 2009). During the biofumigation process release of volatiles suggests that control measures could be used to improve the control of soil-borne diseases (Mercier and Jiménez, 2009). A study in crop fields in Arkansas to investigate the impact of crop rotation and soil cultivation methods on rhizosphere microbial diversity was found not to significantly affect bacterial diversity (Oliveira et al., 2022).

Anaerobic soil disinfestation methods were traditionally used for grasses, but their effect was unknown. Later, it was studied that organic materials, including organic wastes, animal and green manure, compost, and peats, are found to be effective mainly against pests and partially for pathogens (Runia et al., 2012). But these studies showed the effectiveness and production of gasses (CO_2_, CH_4_, and N_2_O, among others) depending on physical factors, including soil type, organic materials, temperature, exposure time, and dosage. Organic amendments included organic wastes, animal and green manure, compost, and peats. In a metastudy, the characteristics of organic soil amendments that improve soil fertility, plant health and suppress soil-borne plant diseases were identified (Bonanomi et al., 2010). Effective treatment against Verticillium wilt in plants through the treatment of organic amendments was done (Vitullo et al., 2013). In short, organic matter possesses positive agronomic properties in agriculture. For the effective soil disinfectant, studies on complete mechanisms that are effective, quick, and predictable are needed.

## Future Trends in Metagenomic Studies

Metagenomic studies itself is a potent tool. However, metagenomics along with wide association studies discussed in this review and transcriptomics studies termed metatranscriptomics will strengthen this approach as a whole. Similarly, proteome study of microbial communities is a new emerging area that aims at analyzing the catalytic potential of a microbial population. Along with agriculture, metagenomics has also been used in food obtained as agriculture byproducts. For example, it is applied in food industries for the identification of novel enzymes and enhances the production of recombinant enzymes (El Sheikha, 2018; Hua et al., 2018). The role of metagenomics in the identification of biocontrol agents as future plant health detectors and influencers in biopolitics and bioeconomics is highly promising for application in near future.

## Conclusion

Enormous studies by a group of researchers from multiple fields have made extraordinary advances in metagenomics over more than three decades. To solve the intricacies of underlying processes of microbial communities, it is important to understand the diversity and dynamics of such microbial communities. This review is a vast resource of knowledge discovery with product discovery. The appreciable NGS cost reduction and community approach can be promising in uncovering the metagenome. Understanding of genome alone of any agricultural microorganism is not enough, as productivity is the cumulative role of genomes rather than the genome of a single organism, be it agricultural crop or fish, or domestic animal. More investment and attention are required to improve the model of genome-wide association studies or genomic selection, adding a metagenome profile in the mixed model. Genome manipulation is inadequate for genetic gain in breeding programs if we neglect the metagenome as one of the major variables. This review highlights the unexplored high potential area, whereas more research attention is required to have better agricultural productivity with sustainability. With the advanced data generation and analyses technologies, this field of metagenomics will widen and improve the knowledge of microorganisms.

## Author Contributions

DK, MI, SJ, and JJ contributed to conceptualization, data curation, formal analysis, funding acquisition, investigation, methodology, project administration, resources, software, supervision, validation, visualization, and writing the original draft. AR, DK, and RP contributed to manuscript editing and finalization. All authors contributed to the article and approved the submitted version.

## Conflict of Interest

The authors declare that the research was conducted in the absence of any commercial or financial relationships that could be construed as a potential conflict of interest.

## Publisher’s Note

All claims expressed in this article are solely those of the authors and do not necessarily represent those of their affiliated organizations, or those of the publisher, the editors and the reviewers. Any product that may be evaluated in this article, or claim that may be made by its manufacturer, is not guaranteed or endorsed by the publisher.

## Footnotes

1
https://www.jcvi.org/research/metarep

2
https://img.jgi.doe.gov/

3
https://gfo.ca/research-projects/w2017id03/

4
https://www.ipcc.ch/srccl/chapter/chapter-5/
